# 2-(3-Nitro­phen­yl)-3-phenyl-2,3-di­hydro-4*H*-1,3-benzo­thia­zin-4-one

**DOI:** 10.1107/S1600536813028389

**Published:** 2013-10-23

**Authors:** Hemant P. Yennawar, Lee J. Silverberg, Michael J. Minehan, John Tierney

**Affiliations:** a Department of Chemistry, Pennsylvania State University, University Park, PA 16802, USA; b Pennsylvania State University, Schuylkill Campus, 200 University Drive, Schuylkill Haven, PA 17972, USA; c Pennsylvania State University, Brandywine Campus, 312 M Main Building, 25 Yearsley Mill Rd., Media, PA 19063, USA

## Abstract

The title compound, C_20_H_14_N_2_O_3_S, has three aromatic rings, *viz.* (i) a phenyl ring, (ii) a 3-nitro­phenyl and (iii) a 1,3-benzo­thia­zine fused-ring system. The dihedral angle between (i) and (ii) is 85.31 (15)°, between (ii) and (iii) is 81.33 (15)° and between (i) and (iii) is 75.73 (15)°. The six-membered 1,3-thia­zine ring has an envelope conformation with the C atom in the 2-position forming the flap. In the crystal, mol­ecules are linked by weak C—H⋯O inter­actions, forming a three-dimensional network.

## Related literature

For amide bond formation using 2,4,6-tripropyl-1,3,5,2,4,6-trioxatri­phospho­rinane-2,4,6-trioxide (T3P), see: Dunetz *et al.* (2011 ▶). For preparation of various heterocycles using imines and T3P, see: Unsworth *et al.* (2013 ▶). For a review of 1,3-thia­zin-4-ones, see: Ryabukhin *et al.* (1996 ▶). For other 2,3-diaryl-2,3-di­hydro-1,3-benzo­thia­zin-4-ones, see: Kamel *et al.* (2010 ▶); Kollenz & Ziegler (1970 ▶); Oae & Numata (1974 ▶); Ponci *et al.* (1963 ▶); Zarghi *et al.* (2009 ▶).
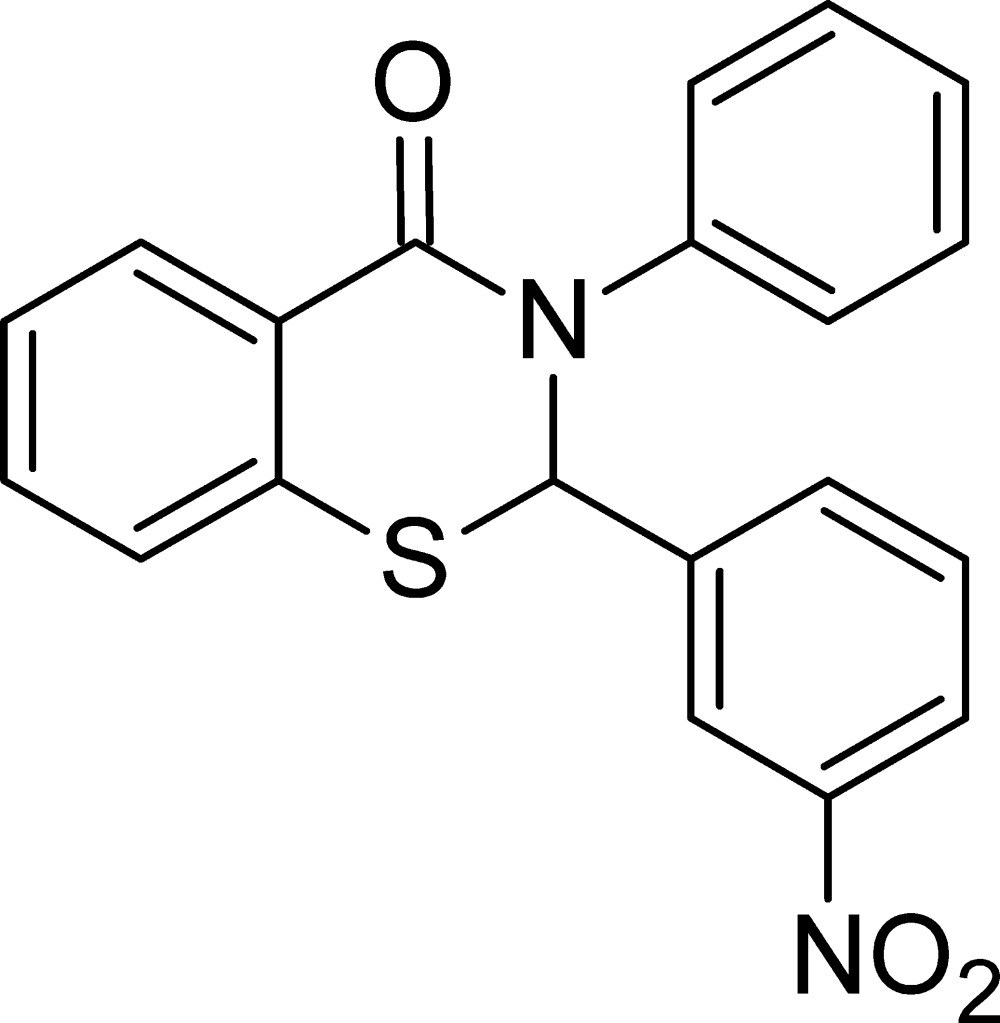


## Experimental

### 

#### Crystal data

C_20_H_14_N_2_O_3_S
*M* *_r_* = 362.39
Monoclinic, 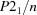

*a* = 9.8741 (13) Å
*b* = 13.0544 (18) Å
*c* = 13.365 (2) Å
β = 100.878 (4)°
*V* = 1691.7 (7) Å^3^
*Z* = 4
Mo *K*α radiation
μ = 0.22 mm^−1^
*T* = 298 K
0.27 × 0.25 × 0.24 mm

#### Data collection

Bruker SMART APEX CCD diffractometer
Absorption correction: multi-scan (*SADABS*; Bruker, 2001 ▶) *T*_min_ = 0.945, *T*_max_ = 0.951
9929 measured reflections
2904 independent reflections
2578 reflections with *I* > 2σ(*I*)
*R*_int_ = 0.023

#### Refinement

*R*[*F* ^2^ > 2σ(*F* ^2^)] = 0.034
*wR*(*F* ^2^) = 0.088
*S* = 1.04
2904 reflections
235 parameters
H-atom parameters not refined
Δρ_max_ = 0.16 e Å^−3^
Δρ_min_ = −2.21 e Å^−3^

### 

Data collection: *SMART* (Bruker, 2001 ▶); cell refinement: *SAINT* (Bruker, 2001 ▶); data reduction: *SAINT*; program(s) used to solve structure: *SHELXS97* (Sheldrick, 2008 ▶); program(s) used to refine structure: *SHELXL97* (Sheldrick, 2008 ▶); molecular graphics: *XSHELL* (Bruker, 2001 ▶); software used to prepare material for publication: *ORTEP-3 for Windows* (Farrugia, 2012 ▶).

## Supplementary Material

Crystal structure: contains datablock(s) I. DOI: 10.1107/S1600536813028389/fy2105sup1.cif

Structure factors: contains datablock(s) I. DOI: 10.1107/S1600536813028389/fy2105Isup2.hkl

Supplementary material file. DOI: 10.1107/S1600536813028389/fy2105Isup3.mol

Supplementary material file. DOI: 10.1107/S1600536813028389/fy2105Isup4.cml

Additional supplementary materials:  crystallographic information; 3D view; checkCIF report

## Figures and Tables

**Table 1:** Hydrogen-bond geometry (Å, °)

*D*—H⋯*A*	*D*—H	H⋯*A*	*D*⋯*A*	*D*—H⋯*A*
C19—H19⋯O3^i^	0.93	2.67	3.334 (8)	129
C7—H7⋯O2^ii^	0.98	2.58	3.424 (8)	145
C11—H11⋯O3^iii^	0.93	2.71	3.362 (6)	128

Symmetry codes: (i) 
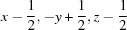
; (ii) 
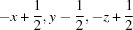
; (iii) 
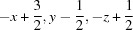
.

**Fig. 1.**
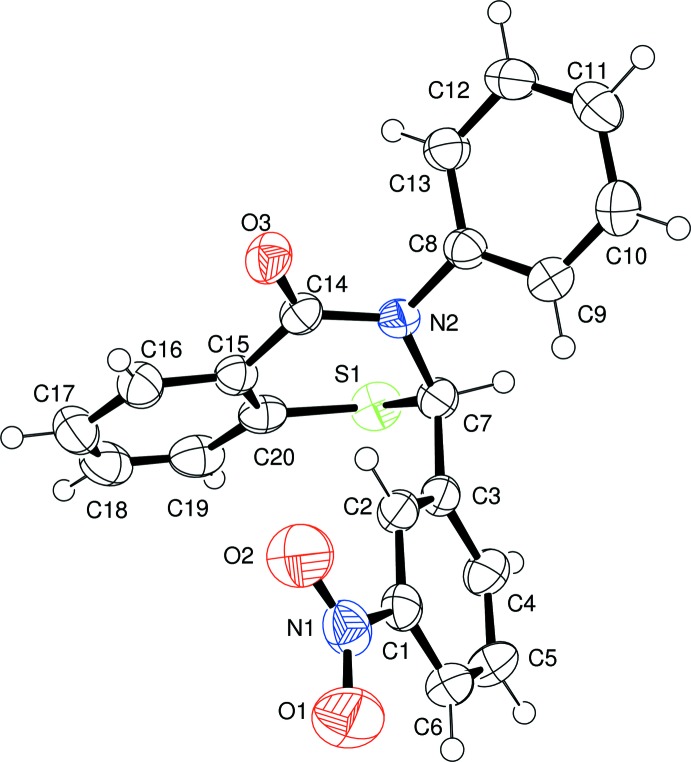
ORTEP view of the title comound. Thermal ellipsoids are drawn at 50% probability.

**Fig. 2.**
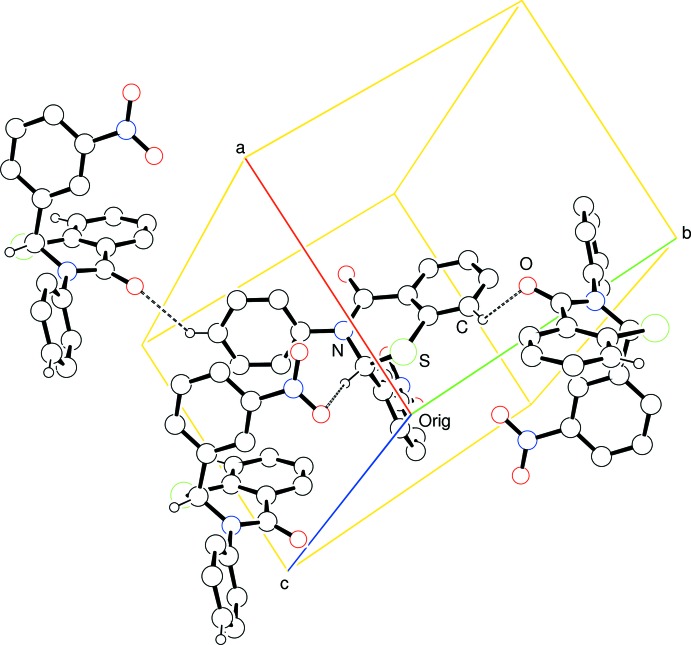
Crystal packing. C—H···O interactions are shown as dashed lines.

## supplementary crystallographic information

### 1. Comment

The six-membered 1,3-thiazin-4-one ring system has often been investigated for its biological activity and is of potential medicinal use (Ryabukhin *et al.*, 1996)). As part of our studies of cyclic 1,3-thiaza-4-one compounds, we wished to form 2,3-diaryl-2,3-dihydro-1,3-benzothiazin-4-ones, of which only a small number have been reported (Kamel *et al.*, 2010; Kollenz *et al.*, 1970; Oae *et al.*, 1974; Ponci *et al.*, 1963; Zarghi *et al.*, 2009). The title molecule was synthesized by condensation of 1-(3-nitrophenyl)-*N*-phenylmethanimine with thiosalicylic acid in the presence of 2,4,6-tripropyl-1,3,5,2,4,6-trioxatriphosphorinane-2,4,6-trioxide (T3P) and pyridine (Dunetz *et al.*, 2011). A similar preparation of a 2,3-dialkyl-2,3-dihydro-1,3-benzothiazin-4-one was recently reported (Unsworth *et al.*, 2013). We report here the crystal structure (Fig. 1) of the title compound, which has three aromatic rings: i) phenyl, ii) nitrophenyl and iii) phenyl, fused to a thiazine ring. The dihedral angle between (i) and (ii) is 85.31 (15)°, between (ii) and (iii) is 81.33 (15)° and between (i) and (iii) is 75.73 (15)°. The thiazine ring has an envelope conformation with the C atom in the 2-position forming the flap. In the crystal packing (Fig. 2), molecules are linked by weak C—H···O interactions (Table 1), resulting in a three-dimensional network.

### 2. Experimental

A two-necked 25 ml roundbottom flask was oven-dried, cooled under N_2_, and charged with a stir bar and 1-(3-nitrophenyl)-*N*-phenylmethanimine (1.3577 g, 6 mmol). Tetrahydrofuran (2.3 ml) was added, the solid dissolved, and the solution was stirred. Pyridine (1.95 ml, 24 mmol) was added and then thiosalicylic acid (0.9311 g, 6 mmol) was added. Finally, 2,4,6-tripropyl-1,3,5,2,4,6-trioxatriphosphorinane-2,4,6-trioxide in 2-methyltetrahydrofuran (50 weight percent; 7.1 ml, 12 mmol) was added. The reaction was stirred at room temperature for 46 h, then poured into a separatory funnel with dichloromethane. The mixture was washed with saturated sodium bicarbonate. The aqueous was then extracted three times with dichloromethane. The organics were combined and washed with saturated sodium bicarbonate, water, and saturated sodium chloride. The organic was dried over sodium sulfate, concentrated *in vacuo* and chromatographed on 30 g flash silica gel, eluting with 20–50% mixtures of ethyl acetate and hexanes. The product eluted with 30–40% EtOAc/hexanes and was concentrated *in vacuo* to a light yellow solid (1.0659 g). Recrystallization from ethyl acetate/hexanes gave light yellow crystals (0.8659 g, 39.8%). m.p.: 163–165°C. R_f_ = 0.62 (50% EtOAc/hexanes). Crystals for X-ray crystallography were grown by slow evaporation from ethanol.

### 3. Refinement

The C-bound H atoms were geometrically placed with C—H = 0.93–0.97 Å, and refined as riding with *U*_iso_(H) = 1.2U_eq_(C).

### Crystal data

**Table:**

C_20_H_14_N_2_O_3_S	*D*_x_ = 1.423 Mg m^−^^3^
*M**_r_* = 362.39	Melting point: 436 K
Monoclinic, *P*2_1_/*n*	Mo *K*α radiation, λ = 0.71073 Å
*a* = 9.8741 (13) Å	Cell parameters from 3778 reflections
*b* = 13.0544 (18) Å	θ = 2.2–28.2°
*c* = 13.365 (2) Å	µ = 0.22 mm^−^^1^
β = 100.878 (4)°	*T* = 298 K
*V* = 1691.7 (4) Å^3^	Block, colorless
*Z* = 4	0.27 × 0.25 × 0.24 mm
*F*(000) = 752	

### Data collection

**Table:**

Bruker SMART-APEX CCD diffractometer	2904 independent reflections
Radiation source: fine-focus sealed tube	2572 reflections with *I* > 2σ(*I*)
Graphite monochromator	*R*_int_ = 0.023
Detector resolution: 8.34 pixels mm^-1^	θ_max_ = 25.0°, θ_min_ = 2.2°
φ and ω scans	*h* = −11→10
Absorption correction: multi-scan (*SADABS*; Bruker, 2001)	*k* = −14→15
*T*_min_ = 0.944, *T*_max_ = 0.950	*l* = −15→15
9929 measured reflections	

### Refinement

**Table:**

Refinement on *F*^2^	Primary atom site location: structure-invariant direct methods
Least-squares matrix: full	Secondary atom site location: difference Fourier map
*R*[*F*^2^ > 2σ(*F*^2^)] = 0.034	Hydrogen site location: inferred from neighbouring sites
*wR*(*F*^2^) = 0.088	H-atom parameters not refined
*S* = 1.04	*w* = 1/[σ^2^(*F*_o_^2^) + (0.0455*P*)^2^ + 0.3773*P*] where *P* = (*F*_o_^2^ + 2*F*_c_^2^)/3
2904 reflections	(Δ/σ)_max_ < 0.001
235 parameters	Δρ_max_ = 0.16 e Å^−^^3^
0 restraints	Δρ_min_ = −0.22 e Å^−^^3^

### Special details

**Table:**

Experimental. The data collection nominally covered a full sphere of reciprocal space by a combination of 4 sets of ω scans each set at different φ and/or 2θ angles and each scan (10 s exposure) covering -0.300° degrees in ω. The crystal to detector distance was 5.82 cm. SADABS was used for absorption correction. R(int) was 0.0320 before and 0.0220 after correction. The Ratio of minimum to maximum transmission is 0.8417. The λ/2 correction factor is 0.0015.
Geometry. All esds (except the esd in the dihedral angle between two l.s. planes) are estimated using the full covariance matrix. The cell esds are taken into account individually in the estimation of esds in distances, angles and torsion angles; correlations between esds in cell parameters are only used when they are defined by crystal symmetry. An approximate (isotropic) treatment of cell esds is used for estimating esds involving l.s. planes.
Refinement. Refinement of F^2^ against ALL reflections. The weighted R-factor wR and goodness of fit S are based on F^2^, conventional R-factors R are based on F, with F set to zero for negative F^2^. The threshold expression of F^2^ > 2sigma(F^2^) is used only for calculating R-factors(gt) etc. and is not relevant to the choice of reflections for refinement. R-factors based on F^2^ are statistically about twice as large as those based on F, and R- factors based on ALL data will be even larger.

### Fractional atomic coordinates and isotropic or equivalent isotropic displacement parameters (Å^2^)

**Table:**

	*x*	*y*	*z*	*U*_iso_*/*U*_eq_	
C1	0.14364 (16)	0.21740 (11)	0.36136 (10)	0.0386 (3)	
C2	0.22760 (15)	0.17463 (11)	0.30037 (10)	0.0360 (3)	
H2	0.3208	0.1912	0.3105	0.043*	
C3	0.17090 (14)	0.10692 (11)	0.22415 (10)	0.0342 (3)	
C4	0.03139 (16)	0.08233 (13)	0.21336 (12)	0.0457 (4)	
H4	−0.0081	0.0371	0.1624	0.055*	
C5	−0.04918 (17)	0.12405 (15)	0.27706 (14)	0.0538 (4)	
H5	−0.1415	0.1054	0.2696	0.065*	
C6	0.00665 (17)	0.19323 (13)	0.35175 (12)	0.0477 (4)	
H6	−0.0471	0.2225	0.3943	0.057*	
C7	0.25569 (14)	0.05832 (12)	0.15304 (10)	0.0362 (3)	
H7	0.2442	−0.0160	0.1579	0.043*	
C8	0.49204 (14)	−0.00021 (11)	0.23007 (10)	0.0346 (3)	
C9	0.45797 (17)	−0.05383 (13)	0.31141 (12)	0.0436 (4)	
H9	0.3771	−0.0386	0.3344	0.052*	
C10	0.54491 (18)	−0.13033 (13)	0.35838 (12)	0.0504 (4)	
H10	0.5214	−0.1671	0.4122	0.060*	
C11	0.66539 (18)	−0.15198 (13)	0.32568 (12)	0.0493 (4)	
H11	0.7248	−0.2019	0.3585	0.059*	
C12	0.69799 (16)	−0.09922 (13)	0.24379 (12)	0.0464 (4)	
H12	0.7790	−0.1147	0.2211	0.056*	
C13	0.61167 (16)	−0.02387 (12)	0.19526 (11)	0.0402 (3)	
H13	0.6338	0.0107	0.1396	0.048*	
C14	0.45482 (14)	0.17527 (11)	0.16948 (10)	0.0345 (3)	
C15	0.36533 (15)	0.24938 (11)	0.10216 (10)	0.0357 (3)	
C16	0.40628 (17)	0.35182 (12)	0.10646 (12)	0.0445 (4)	
H16	0.4810	0.3725	0.1556	0.053*	
C17	0.3380 (2)	0.42271 (14)	0.03930 (13)	0.0535 (4)	
H17	0.3657	0.4909	0.0437	0.064*	
C18	0.2281 (2)	0.39235 (15)	−0.03486 (13)	0.0560 (5)	
H18	0.1839	0.4399	−0.0818	0.067*	
C19	0.18368 (17)	0.29236 (15)	−0.03962 (11)	0.0502 (4)	
H19	0.1089	0.2728	−0.0892	0.060*	
C20	0.25026 (15)	0.22016 (12)	0.02946 (10)	0.0393 (4)	
N1	0.20361 (17)	0.29450 (10)	0.43715 (10)	0.0481 (3)	
N2	0.40305 (12)	0.07868 (9)	0.17982 (9)	0.0355 (3)	
O1	0.12985 (17)	0.33388 (12)	0.48963 (10)	0.0775 (4)	
O2	0.32476 (15)	0.31730 (11)	0.44327 (11)	0.0690 (4)	
O3	0.57097 (11)	0.19924 (8)	0.21252 (8)	0.0449 (3)	
S1	0.19079 (4)	0.09311 (3)	0.02046 (3)	0.04701 (15)	

### Atomic displacement parameters (Å^2^)

**Table:**

	*U*^11^	*U*^22^	*U*^33^	*U*^12^	*U*^13^	*U*^23^
C1	0.0458 (9)	0.0348 (8)	0.0366 (7)	0.0022 (6)	0.0111 (6)	0.0069 (6)
C2	0.0328 (7)	0.0382 (8)	0.0374 (7)	−0.0008 (6)	0.0076 (6)	0.0051 (6)
C3	0.0313 (7)	0.0341 (8)	0.0371 (7)	−0.0008 (6)	0.0063 (6)	0.0042 (6)
C4	0.0368 (8)	0.0472 (10)	0.0530 (9)	−0.0085 (7)	0.0088 (7)	−0.0021 (7)
C5	0.0349 (8)	0.0632 (11)	0.0672 (11)	−0.0059 (8)	0.0193 (8)	0.0036 (9)
C6	0.0474 (9)	0.0488 (10)	0.0524 (9)	0.0053 (8)	0.0236 (7)	0.0068 (7)
C7	0.0315 (8)	0.0348 (8)	0.0413 (7)	−0.0033 (6)	0.0041 (6)	−0.0027 (6)
C8	0.0318 (7)	0.0348 (8)	0.0365 (7)	0.0003 (6)	0.0050 (6)	−0.0026 (6)
C9	0.0398 (8)	0.0447 (9)	0.0491 (8)	0.0002 (7)	0.0158 (7)	0.0032 (7)
C10	0.0570 (10)	0.0462 (10)	0.0491 (9)	−0.0009 (8)	0.0130 (8)	0.0109 (7)
C11	0.0498 (10)	0.0430 (10)	0.0522 (9)	0.0100 (8)	0.0020 (7)	0.0033 (7)
C12	0.0369 (8)	0.0503 (10)	0.0525 (9)	0.0082 (7)	0.0102 (7)	−0.0031 (7)
C13	0.0384 (8)	0.0446 (9)	0.0387 (7)	0.0032 (7)	0.0101 (6)	0.0016 (6)
C14	0.0341 (8)	0.0385 (8)	0.0316 (7)	−0.0021 (6)	0.0082 (6)	−0.0016 (6)
C15	0.0362 (8)	0.0400 (9)	0.0323 (7)	0.0016 (6)	0.0103 (6)	−0.0007 (6)
C16	0.0483 (9)	0.0422 (9)	0.0445 (8)	0.0014 (7)	0.0126 (7)	−0.0003 (7)
C17	0.0640 (11)	0.0416 (10)	0.0594 (10)	0.0098 (8)	0.0227 (9)	0.0079 (7)
C18	0.0591 (11)	0.0615 (12)	0.0504 (9)	0.0228 (9)	0.0185 (8)	0.0185 (8)
C19	0.0417 (9)	0.0724 (13)	0.0366 (8)	0.0123 (8)	0.0071 (7)	0.0065 (7)
C20	0.0358 (8)	0.0519 (10)	0.0315 (7)	0.0048 (7)	0.0101 (6)	−0.0012 (6)
N1	0.0650 (10)	0.0413 (8)	0.0393 (7)	0.0036 (7)	0.0134 (7)	0.0025 (6)
N2	0.0288 (6)	0.0365 (7)	0.0411 (6)	0.0009 (5)	0.0064 (5)	0.0003 (5)
O1	0.0956 (11)	0.0797 (10)	0.0658 (8)	0.0015 (8)	0.0373 (8)	−0.0239 (7)
O2	0.0631 (9)	0.0687 (9)	0.0741 (9)	−0.0121 (7)	0.0103 (7)	−0.0241 (7)
O3	0.0368 (6)	0.0479 (7)	0.0465 (6)	−0.0081 (5)	−0.0013 (5)	0.0042 (5)
S1	0.0444 (2)	0.0576 (3)	0.0362 (2)	−0.00628 (18)	0.00036 (17)	−0.00963 (16)

### Geometric parameters (Å, º)

**Table:**

C1—C6	1.371 (2)	C11—C12	1.382 (2)
C1—C2	1.385 (2)	C11—H11	0.9300
C1—N1	1.471 (2)	C12—C13	1.381 (2)
C2—C3	1.385 (2)	C12—H12	0.9300
C2—H2	0.9300	C13—H13	0.9300
C3—C4	1.395 (2)	C14—O3	1.2227 (17)
C3—C7	1.519 (2)	C14—N2	1.3773 (19)
C4—C5	1.382 (2)	C14—C15	1.492 (2)
C4—H4	0.9300	C15—C16	1.395 (2)
C5—C6	1.381 (3)	C15—C20	1.401 (2)
C5—H5	0.9300	C16—C17	1.374 (2)
C6—H6	0.9300	C16—H16	0.9300
C7—N2	1.4558 (18)	C17—C18	1.383 (3)
C7—S1	1.8242 (14)	C17—H17	0.9300
C7—H7	0.9800	C18—C19	1.375 (3)
C8—C13	1.384 (2)	C18—H18	0.9300
C8—C9	1.387 (2)	C19—C20	1.394 (2)
C8—N2	1.4356 (18)	C19—H19	0.9300
C9—C10	1.387 (2)	C20—S1	1.7560 (17)
C9—H9	0.9300	N1—O1	1.2163 (19)
C10—C11	1.372 (2)	N1—O2	1.2203 (19)
C10—H10	0.9300		
			
C6—C1—C2	122.79 (15)	C12—C11—H11	120.2
C6—C1—N1	118.93 (14)	C13—C12—C11	120.79 (15)
C2—C1—N1	118.24 (14)	C13—C12—H12	119.6
C3—C2—C1	119.11 (14)	C11—C12—H12	119.6
C3—C2—H2	120.4	C12—C13—C8	119.45 (14)
C1—C2—H2	120.4	C12—C13—H13	120.3
C2—C3—C4	118.50 (14)	C8—C13—H13	120.3
C2—C3—C7	122.20 (13)	O3—C14—N2	121.34 (13)
C4—C3—C7	119.29 (13)	O3—C14—C15	120.92 (13)
C5—C4—C3	121.17 (15)	N2—C14—C15	117.73 (12)
C5—C4—H4	119.4	C16—C15—C20	118.70 (14)
C3—C4—H4	119.4	C16—C15—C14	117.55 (13)
C6—C5—C4	120.34 (15)	C20—C15—C14	123.51 (14)
C6—C5—H5	119.8	C17—C16—C15	121.06 (16)
C4—C5—H5	119.8	C17—C16—H16	119.5
C1—C6—C5	118.05 (15)	C15—C16—H16	119.5
C1—C6—H6	121.0	C16—C17—C18	119.82 (17)
C5—C6—H6	121.0	C16—C17—H17	120.1
N2—C7—C3	114.41 (11)	C18—C17—H17	120.1
N2—C7—S1	110.18 (10)	C19—C18—C17	120.38 (16)
C3—C7—S1	111.69 (10)	C19—C18—H18	119.8
N2—C7—H7	106.7	C17—C18—H18	119.8
C3—C7—H7	106.7	C18—C19—C20	120.32 (16)
S1—C7—H7	106.7	C18—C19—H19	119.8
C13—C8—C9	119.98 (14)	C20—C19—H19	119.8
C13—C8—N2	119.27 (13)	C19—C20—C15	119.63 (15)
C9—C8—N2	120.74 (13)	C19—C20—S1	118.90 (12)
C8—C9—C10	119.81 (15)	C15—C20—S1	121.43 (11)
C8—C9—H9	120.1	O1—N1—O2	122.98 (15)
C10—C9—H9	120.1	O1—N1—C1	118.64 (16)
C11—C10—C9	120.25 (15)	O2—N1—C1	118.37 (14)
C11—C10—H10	119.9	C14—N2—C8	119.85 (11)
C9—C10—H10	119.9	C14—N2—C7	121.01 (12)
C10—C11—C12	119.69 (15)	C8—N2—C7	118.63 (12)
C10—C11—H11	120.2	C20—S1—C7	96.72 (7)
			
C6—C1—C2—C3	2.1 (2)	C16—C17—C18—C19	2.2 (3)
N1—C1—C2—C3	−175.83 (12)	C17—C18—C19—C20	−0.9 (3)
C1—C2—C3—C4	−1.6 (2)	C18—C19—C20—C15	−1.8 (2)
C1—C2—C3—C7	178.93 (13)	C18—C19—C20—S1	−179.74 (12)
C2—C3—C4—C5	−0.1 (2)	C16—C15—C20—C19	3.0 (2)
C7—C3—C4—C5	179.38 (15)	C14—C15—C20—C19	−171.23 (13)
C3—C4—C5—C6	1.5 (3)	C16—C15—C20—S1	−179.07 (11)
C2—C1—C6—C5	−0.7 (2)	C14—C15—C20—S1	6.68 (19)
N1—C1—C6—C5	177.16 (14)	C6—C1—N1—O1	0.6 (2)
C4—C5—C6—C1	−1.0 (3)	C2—C1—N1—O1	178.58 (14)
C2—C3—C7—N2	7.31 (19)	C6—C1—N1—O2	−178.18 (14)
C4—C3—C7—N2	−172.17 (13)	C2—C1—N1—O2	−0.2 (2)
C2—C3—C7—S1	−118.72 (13)	O3—C14—N2—C8	−8.7 (2)
C4—C3—C7—S1	61.80 (16)	C15—C14—N2—C8	170.05 (12)
C13—C8—C9—C10	0.7 (2)	O3—C14—N2—C7	162.90 (13)
N2—C8—C9—C10	179.50 (14)	C15—C14—N2—C7	−18.30 (18)
C8—C9—C10—C11	1.0 (2)	C13—C8—N2—C14	−55.44 (18)
C9—C10—C11—C12	−1.8 (3)	C9—C8—N2—C14	125.78 (15)
C10—C11—C12—C13	0.9 (3)	C13—C8—N2—C7	132.72 (14)
C11—C12—C13—C8	0.8 (2)	C9—C8—N2—C7	−46.06 (18)
C9—C8—C13—C12	−1.6 (2)	C3—C7—N2—C14	−69.47 (16)
N2—C8—C13—C12	179.58 (13)	S1—C7—N2—C14	57.35 (15)
O3—C14—C15—C16	−12.9 (2)	C3—C7—N2—C8	102.28 (14)
N2—C14—C15—C16	168.26 (13)	S1—C7—N2—C8	−130.90 (11)
O3—C14—C15—C20	161.39 (14)	C19—C20—S1—C7	−155.36 (12)
N2—C14—C15—C20	−17.4 (2)	C15—C20—S1—C7	26.72 (13)
C20—C15—C16—C17	−1.7 (2)	N2—C7—S1—C20	−54.94 (11)
C14—C15—C16—C17	172.90 (14)	C3—C7—S1—C20	73.38 (11)
C15—C16—C17—C18	−0.9 (2)		

### Hydrogen-bond geometry (Å, º)

**Table:**

*D*—H···*A*	*D*—H	H···*A*	*D*···*A*	*D*—H···*A*
C19—H19···O3^i^	0.93	2.63	3.2912 (19)	129
C7—H7···O2^ii^	0.98	2.59	3.435 (2)	145
C11—H11···O3^iii^	0.93	2.71	3.363 (2)	128

Symmetry codes: (i) *x*−1/2, −*y*+1/2, *z*−1/2; (ii) −*x*+1/2, *y*−1/2, −*z*+1/2; (iii) −*x*+3/2, *y*−1/2, −*z*+1/2.
